# An Unusual Presentation of Lichen Planus

**DOI:** 10.7759/cureus.19304

**Published:** 2021-11-06

**Authors:** Pranjali Sharma

**Affiliations:** 1 Endocrinology, Parkview Medical Center, Pueblo, USA

**Keywords:** lichen planus pemphigoides, systemic steroids, vesiculobullous skin lesions, pyoderma gangrenosum, atypical lichen planus

## Abstract

Lichen planus is a chronic papulosquamous eruption of the skin, scalp, nails, and mucous membranes. "Pruritic, purple, polygonal, planar, papules, plaques" are the traditional six "P's" of lichen planus. We describe an unusual case of lichen planus presenting as cellulitis.

A 64-year-old lady with a past medical history of pyoderma gangrenosum, inclusion body myositis, and chronic kidney disease presented with a two-week history of swelling, erythema, tenderness, hyperkeratotic plaques, and blisters on the medial aspect of both thighs. She had a previous history of pyoderma gangrenosum exacerbations with similar presentations; however, current lesions were different from prior presentations. We considered the differential diagnoses of bacterial cellulitis versus pyoderma gangrenosum exacerbation. Due to the difference in these lesions from previous episodes, the patient was empirically treated for bacterial cellulitis with intravenous cefepime and linezolid. The infectious diseases team was consulted and valacyclovir was added to cover for possible herpes infection, with no improvement in symptomatology. Dermatology was then consulted, and a clinical diagnosis of psoriasiform dermatitis was made. A skin biopsy was obtained and the patient was started on prednisone. There was an immediate improvement in the papules within 24 hours. The papules cleared, leaving behind violaceous flat plaques, clinically diagnosed as lichen planus. The affected area was shrinking as compared to previous examinations. The skin biopsy was reported as chronic psoriasiform dermatitis with the main differential of lichen planus. The patient was discharged home on a tapering dose of oral prednisone, topical clobetasol, and oral moxifloxacin.

This case demonstrates the importance of familiarity with rare clinical subtypes as a suspicion for lichen planus. The vesiculobullous subtype of lichen planus, as seen in this patient, tends to present as blisters and cellulitis from infection of the bullae. Treatment of the infection alone is not enough and steroids are essential. This knowledge helps change management, allows for earlier improvement and better patient outcomes.

## Introduction

Lichen planus is a chronic papulosquamous eruption of the skin, scalp, nails, and mucous membranes. "Pruritic, purple, polygonal, planar, papules, plaques" are the traditional six "P's" of lichen planus. The papules are usually dry and shiny with scales that form whitish streaks called Wickham's striae. Lesions are commonly bilateral and symmetric. Lichen planus frequently involves the flexor surfaces of extremities, as well as the trunk and sacral regions. Other areas such as the skin, nails, hair, genitals, oral mucosa, esophagus, and conjunctiva may be infrequently affected [1].

Classic forms of lichen planus as described above are more common than other variants of lichen planus. Therefore, unfamiliarity with the variants and their atypical presentation can lead to a delay in diagnosis and can make management difficult. Here, we present a case of vesiculobullous lichen planus that was initially thought to be cellulitis and treated as the same until the appropriate diagnosis was made.

## Case presentation

**Figure 1:**
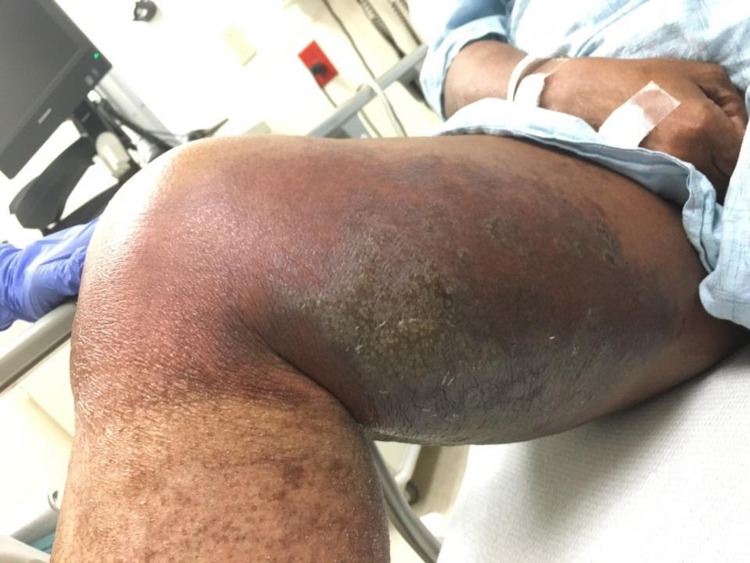
Right thigh at presentation Lesions on the right thigh show hyperkeratotic plaques and blisters in the center with erythema in the periphery. The entire area was tender to touch.

**Figure 2:**
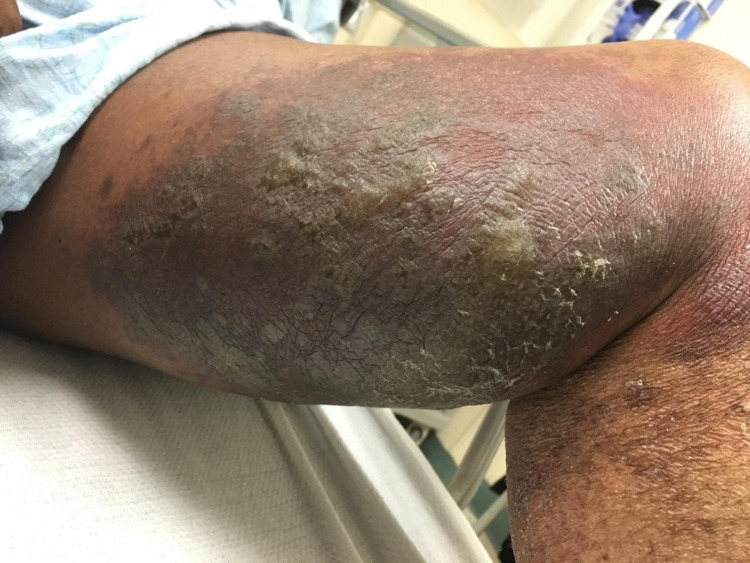
Left thigh at presentation The left thigh shows the hyperkeratotic plaques and blisters more clearly with surrounding erythema. The entire area was also tender to touch.

A 64-year-old woman with a past medical history of pyoderma gangrenosum, inclusion body myositis, and chronic kidney disease presented with hyperkeratotic plaques and blisters on the medial aspect of both thighs for two weeks. The plaques were associated with swelling, erythema, and significant tenderness (Figures 1, 2). 

The patient had a previous history of pyoderma gangrenosum exacerbations with similar presentations that had primarily been treated with topical steroids and intravenous methylprednisolone and cyclosporine when she was previously inpatient. Current lesions, however, were different from prior presentations and associated with a higher degree of pain and tenderness.

On examination, vital signs showed a temperature of 99°F, heart rate of 100 beats/min, and blood pressure of 134/68 mm Hg. Lab evaluation showed neutrophilic leucocytosis on labs (white blood cell {WBC} count was 12,000/ul with 80% neutrophils). The sedimentation rate was elevated at 45 mm/h. Blood cultures were drawn. Due to concern for the immunocompromised state from prior exposure to steroids, cyclosporine, and the clinical suspicion for bacterial cellulitis, the patient received intravenous cefepime and linezolid. After consultation with the infectious disease team, herpes culture was drawn and oral valacyclovir was added for herpes infection.

By day three, there was no change in the appearance of the lesions. We consulted the dermatology team to evaluate for a possible dermatological cause of the patient's lesions. A skin biopsy was performed by them. On day four, blood and herpes cultures had both been negative for 96 hours. By day five, we had ruled out infection as a possible etiology and a clinical suspicion for psoriasiform dermatitis was raised. The skin biopsy confirmed psoriasiform dermatitis with a main differential of lichen planus. Topical clobetasol and oral prednisone 60 mg daily were initiated.

**Figure 3:**
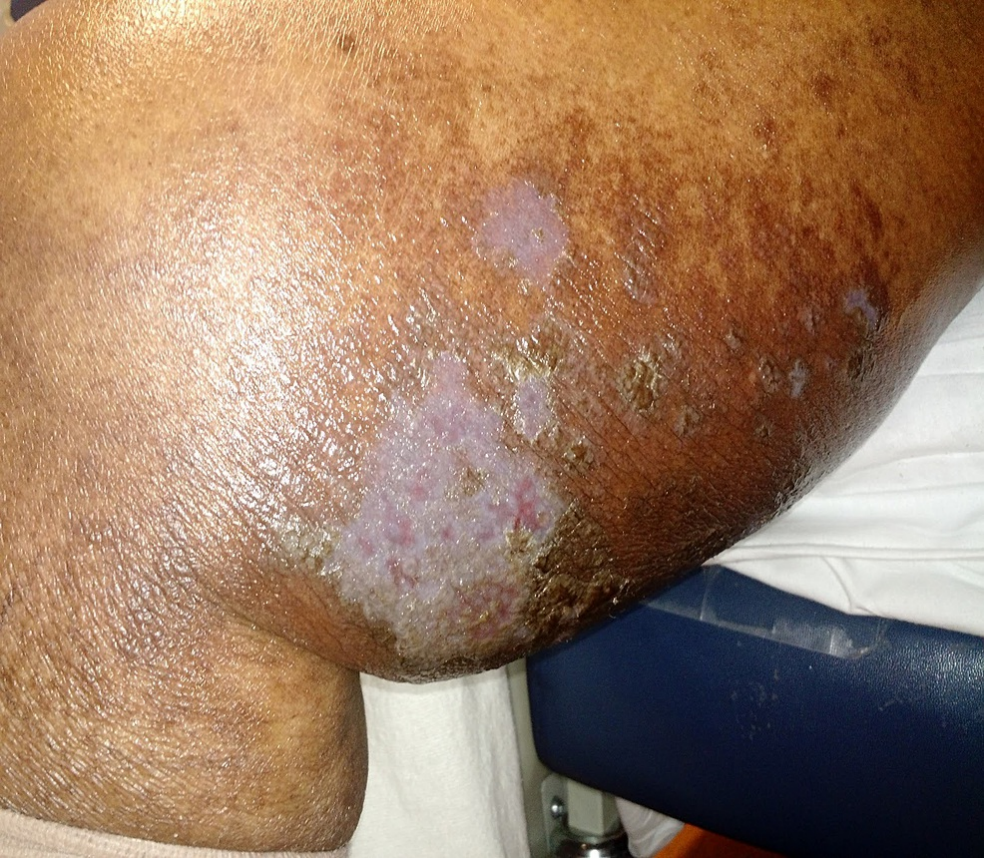
Right thigh 48 hours after steroid initiation The significant improvement and almost complete resolution of the previously noted plaques and blisters and the surrounding erythema. The typical violaceous and polygonal lesions of LP are now more clearly defined. LP: lichen planus

**Figure 4:**
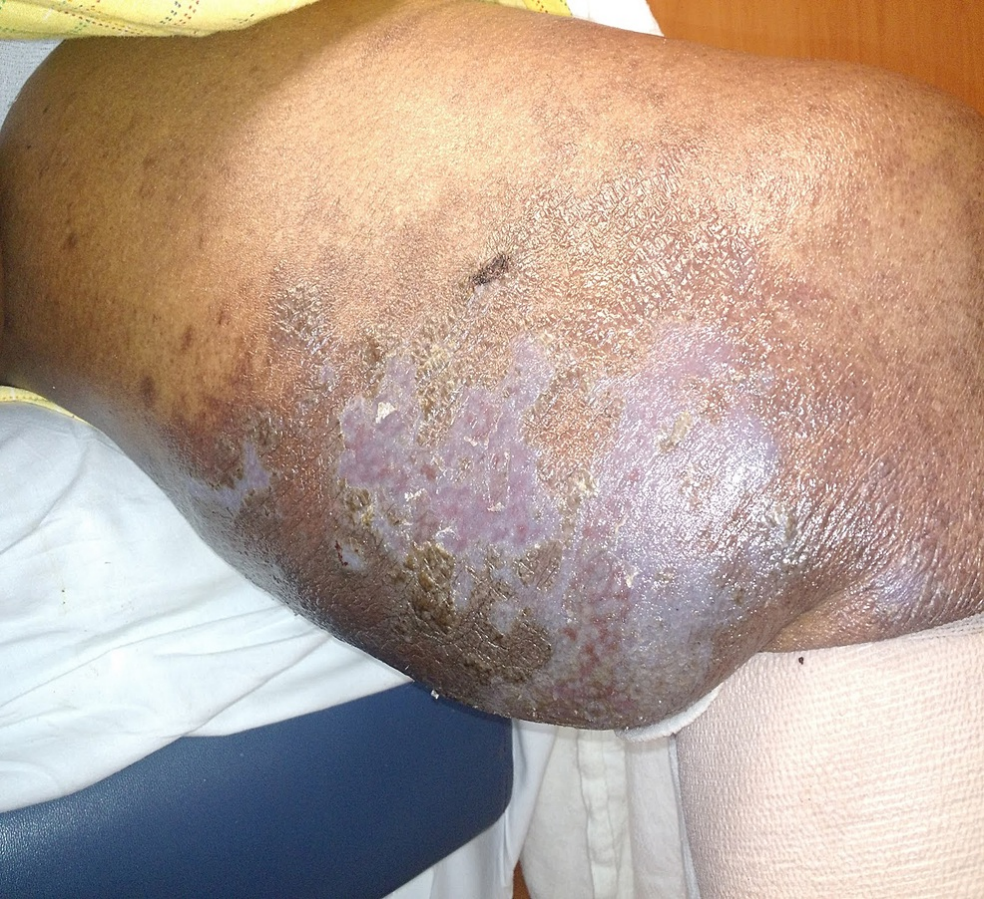
Left thigh 48 hours after steroid initiation The typical violaceous and polygonal lesions of LP are now more clearly defined on the left thigh with a silvery sheen. Erythema has also been resolved. LP: lichen planus

Within 48 hours of prednisone initiation, clinical improvement was noted and the characteristic violaceous plaques were seen on examination (Figures 3, 4). The presence of erythema and discharge from some parts of the lesions suggested possible ongoing cellulitis. The patient was discharged home on oral prednisone taper and topical clobetasone for lichen planus and another seven-day course of moxifloxacin for cellulitis. Four weeks later, significant improvement in the patient's lesions was reported in the dermatology outpatient visit and the oral and topical steroids were discontinued.

## Discussion

Lichen planus is a dermatological condition that occurs in 0.5-1% of the general population [1]. It occurs predominantly in females with a female:male ratio of 4:1 between the third and seventh decades. Anxiety and psychological stress are the main triggering factors for lichen planus. Systemic medications such as beta-blockers, nonsteroidal anti-inflammatory drugs (NSAIDs), diuretics, anti-malarials, sulfonylureas, and dental amalgam are known to exacerbate the lesions. Hepatitis C and chronic liver disease, as well as interferon and ribavirin therapies used to treat Hepatitis C, aggravate lichen planus. Tobacco chewing is associated with oral lichen planus [2].

While classic lichen planus lesions are the most common type of lichen planus, other variants also exist based on morphology and location. These include annular, linear, atrophic, hypertrophic, inverse, eruptive, bullous, ulcerative, lichen planus pigmentosus, lichen planopilaris, vulvovaginal, actinic, lichen planus-lupus erythematosus overlap syndrome, and lichen plan pemphigoides [1].

The bullous form of lichen planus is rare and is characterized by the presence of a vesiculobullous lesion, like that seen in our patient. The typical violaceous and polygonal lesions are hidden by tense bullae making the clinical diagnosis harder [1]. Bullae formation occurs due to vacuolar change of the basement membrane layer [3]. Legs are the most common sites, as in our patient [1]. Other areas where this variant can occur are the hands, feet, and trunk [1]. It commonly presents as cellulitis with infected blisters within plaques. Vesiculobullous lichen planus has to be distinguished from lichen planus pemphigoides through a histological examination [3]. Unfortunately, we did not have access to the skin biopsy images to review the histological findings in our patient due to restricted access to testing done by the private dermatology group.

There is no established or clearly effective treatment of choice for bullous lichen planus. Topical high potency steroids such as betamethasone are used. Systemic glucocorticoids are considered second-line therapy and in severe or refractory cases [4]. There is some literature to support the use of dapsone [5], mycophenolate [6], and retinoic acid [7].

Most cases of cutaneous lichen planus will remit within one to two years [8]. It is unclear if ongoing topical steroid treatment after the resolution of cutaneous lesions prevents future recurrences. Our patient had good clinical improvement after treatment with oral prednisone and topical clobetasone, following which the therapy was ultimately discontinued. However, we do not know if she had any future lichen planus recurrences.

## Conclusions

This case demonstrates the importance of familiarity with the rarer types of lichen planus. Bullous lichen planus is rare and the bullae hide the typical violaceous and polygonal lesions. Therefore, it is easily misdiagnosed as in the case above. Our patient did not present with the typical signs of sepsis one would expect from cellulitis of this degree. She also did not respond to intravenous antibiotics in a typical fashion. This indicated that there was another process causing the lesions. Familiarity with lichen planus and variants would have led to a prompt biopsy, an appropriate diagnosis, and faster improvement in this patient's cutaneous condition. An accurate early diagnosis could also have allowed for appropriate antibiotic stewardship. We suggest that alternate rare diagnoses be considered when the clinical presentation does not follow the expected course of recovery.

## References

[1] Weston G, Payette M (2015). Update on lichen planus and its clinical variants. Int J Womens Dermatol.

[2] Krupaa RJ, Sankari SL, Masthan KM, Rajesh E (2015). Oral lichen planus: an overview. J Pharm Bioallied Sci.

[3] Gawkrodger DJ, Stavropoulos PG, McLaren KM, Buxton PK (1989). Bullous lichen planus and lichen planus pemphigoides--clinico-pathological comparisons. Clin Exp Dermatol.

[4] Liakopoulou A, Rallis E (2017). Bullous lichen planus - a review. J Dermatol Case Rep.

[5] Camisa C, Neff JC, Rossana C, Barrett JL (1986). Bullous lichen planus: diagnosis by indirect immunofluorescence and treatment with dapsone. J Am Acad Dermatol.

[6] Nousari HC, Goyal S, Anhalt GJ (1999). Successful treatment of resistant hypertrophic and bullous lichen planus with mycophenolate mofetil. Arch Dermatol.

[7] van Tuyll van Serooskerken AM, van Marion AM, de Zwart-Storm E, Frank J, Poblete-Gutiérrez P (2007). Lichen planus with bullous manifestation on the lip. Int J Dermatol.

[8] Irvine C, Irvine F, Champion RH (1991). Long-term follow-up of lichen planus. Acta Derm Venereol.

